# Climate Change And Its Impact On Asthma In South Africa

**Published:** 2025-09

**Authors:** Caryn M Upton, Jonny Peter

**Affiliations:** University of Cape Town Lung Institute, South Africa

**Keywords:** climate change, asthma, aeroallergens, air pollution, sentinel surveillance

## Abstract

South Africa is experiencing climate-related warming 1.5 times faster than the global average, which is driving shifts in meteorological and environmental conditions that exacerbate respiratory health risks. More extreme weather events, including heatwaves, floods and wildfires, combined with increasing aeroallergens, mould growth and poor air quality, are contributing to increased rates of asthma and allergic respiratory diseases, at the same time driving morbidity and mortality. While these risks are real and growing, they also present an opportunity to strengthen climate–health resilience. Existing health and environmental monitoring systems remain fragmented and unevenly distributed. Linking environmental exposure data to respiratory health outcomes is essential if public health planning and adaptation are to be effective. This narrative review highlights and contextualises the current evidence on climate-related respiratory risks in South Africa, with a focus on asthma. It highlights recent national studies, identifies key data and policy gaps and introduces SA-CARES, a sentinel-based early-warning system for respiratory health as a model for pre-emptive integrated surveillance. Coordinated investment in data integration, healthcare preparedness and community engagement will be key to building adaptive capacity and advancing climate-resilient health policy in South Africa and other low- and middle-income countries.

## Introduction

Climate change poses the most significant risk to global health today.^1^ In 2023, record levels of key greenhouse gases were reported.^2^ This was followed by the highest global mean temperature in 2024, recorded at 1.55 °C above the 1850–1900 average. It marks the first year on record to surpass the 1.5 °C threshold relative to the pre-industrial period set by the Paris Climate Accord, a threshold for assessing long-term climate risk.^3^ South Africa is experiencing climate-related warming at a rate 1.5 times faster than the global average, intensifying the risks to respiratory health.^4^ The country faces a convergence of infrastructural and environmental vulnerabilities, including poverty, urbanisation, the high prevalence of communicable disease, smoking, indoor air pollution and fragmented healthcare systems that compound the burden of asthma and related respiratory diseases. Climate change exacerbates both allergic and non-allergic respiratory disease through:

- rising temperatures;
- earlier or extended pollen seasons;
- increased ozone and dust exposure; and
- more frequent extreme weather events such as floods and wildfires.^5^

**Figure 1:**
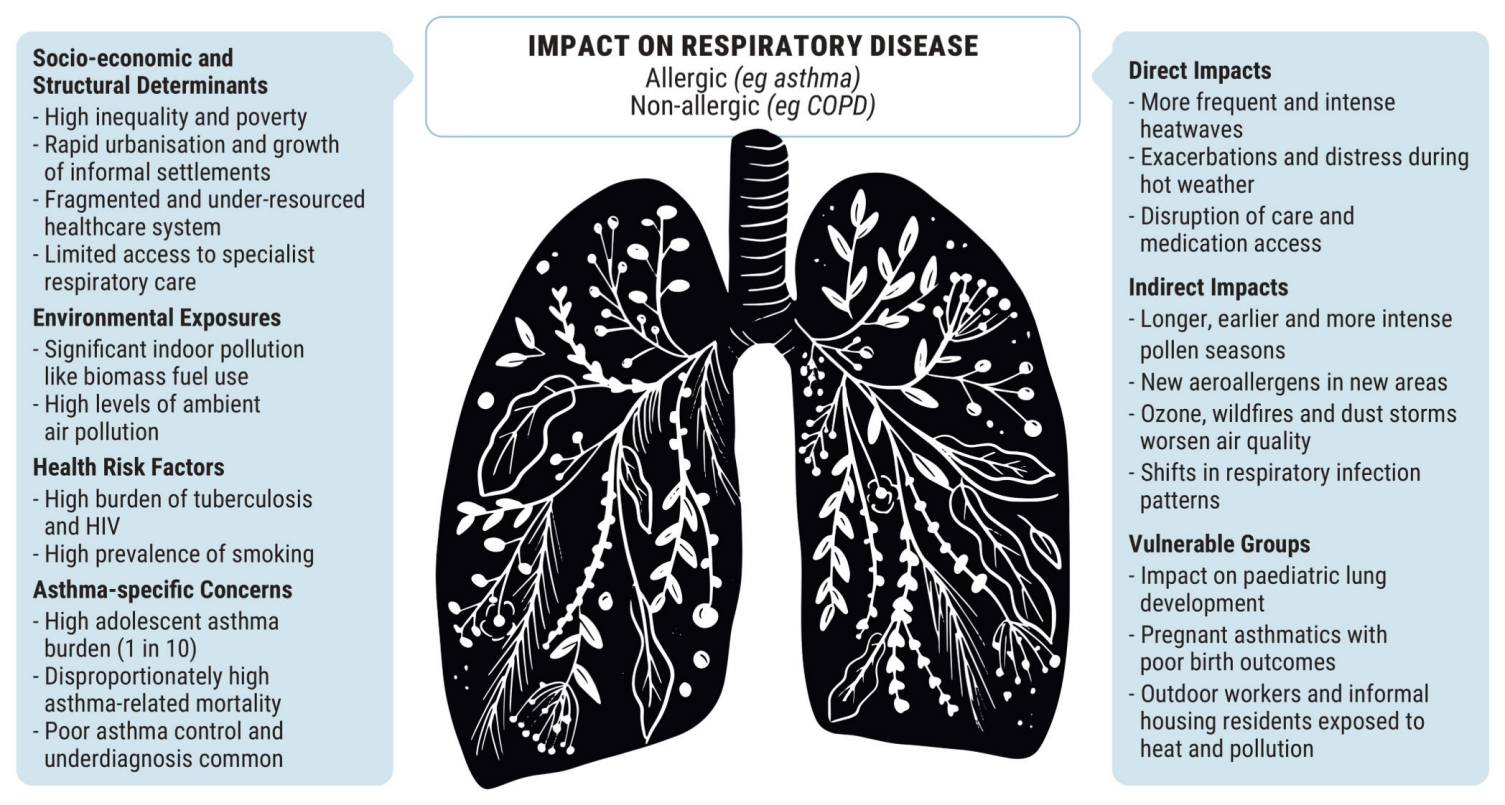
Social and environmental determinants of asthma vulnerability in South Africa’s changing climate

Vulnerable populations, particularly adolescents, children and pregnant women, bear a disproportionate burden. Figure 1 maps the intersection of social and environmental risks that shape respiratory vulnerability, limiting adaptive capacity in South Africa’s changing climate.

### Climate-Linked Exposures And Health Risks

The immediate, widespread and persistent harm caused by climate change across all ages as evidenced by worsening chronic conditions, increased hospitalisations and strained healthcare systems makes respiratory health a key climate-related health concern.^5^ This brief narrative review discusses the main environmental and climatic factors influencing asthma in South Africa. It highlights gaps in local evidence and surveillance systems and explores emerging solutions such as sentinel surveillance and early-warning systems to inform public-health responses and policy in South Africa and other low- and middle-income countries (LMICs).

Climate change drives long-term shifts in meteorological factors at different time scales, including rising surface temperatures on land and at sea, increased climatic variability and more frequent extreme weather events such as heatwaves, cold snaps and flooding. These shifts trigger downstream environmental effects such as soil degradation, crop failure, the spread of invasive species, and extended and more intense pollen seasons.^6^ Warmer, wetter conditions have also enabled the spread of vector-borne diseases such as malaria and tick-borne infections and may facilitate the emergence of new respiratory pathogens.^7,8^

In South Africa, a longitudinal analysis (1920–2023) found a significant increase (*p* < 0.01) in the frequency of floods, tornadoes, snow, strong winds and hailstorms.^9^ In recent years the country has endured successive extreme weather events: multi-year droughts in the Eastern and Northern Cape (2015–2018) devastated agriculture; the Western Cape’s ‘Day Zero’ crisis highlighted water scarcity; and flash floods in KwaZulu-Natal in 2022 caused widespread damage.^9,10^ Several of these extreme weather events have been shown to have had a negative impact on allergic and non-allergic respiratory illness. Heavy rainfall, flooding, high humidity and prolonged damp conditions create an ideal environment for indoor mould growth, worsening allergic rhinitis (AR), asthma and fungal respiratory infections.^11–13^ Nearly 80% of individuals in a post-hurricane cohort reported respiratory issues, including wheezing and breathlessness, which underscored the need for effective post-flood clean-up strategies.^14,15^ Hot and humid conditions elevate ground-level ozone, while cold spells can trigger asthma exacerbations.^5,16^ Temperature variability, not just rising temperatures, has a direct impact on respiratory health. One study found that moderate and extreme cold were responsible for 22% and 2% of asthma-related deaths, respectively, although a recent meta-analysis found the relative risk of cold-related asthma hospitalisations to be non-significant.^16,17^ Although the molecular mechanisms linking temperature to asthma remain poorly understood, in vivo and in vitro models suggest that extremes (10 °C and 40 °C) can impair lung function, increase pro-inflammatory cytokines and lead to airway remodelling and subepithelial fibrosis.^18,19^

Wildfire risk increases with heat and low humidity. Wildfire smoke contains particulate matter (PM_x_), carbon monoxide (CO), nitrogen oxides (NO_x_) and volatile organic compounds.^20^ PM is linked to poor respiratory outcomes.^21^ A Californian study found that wildfire-specific PM_2.5_ was associated with a 30% increase in childhood respiratory admissions, tenfold higher than PM_2.5_ from other sources.^22^ In South Africa, wildfire risk is projected to rise due to increasing vegetation density and reduced rainfall, with an estimated 40 000 wildfires already reported annually in South Africa.^23,24^ Catastrophic fires in the Knysna (2017) and Cape Town (2021) areas caused significant damage to infrastructure. Both ambient and indoor air pollution have a negative impact on respiratory health, and rising temperatures worsen air quality by boosting ozone and contributing to air stagnation.

Aeroallergen monitoring is crucial to the surveillance of respiratory risk. Climate change, via higher temperatures and elevated CO_2_, can increase certain pollen production and extend allergy seasons.^25^ For instance, the highly allergenic ragweed pollen has been extending its range with rising global temperatures, with resultant increases in new sensitisations. We have recently highlighted the detection of Ambrosia species in our northern spore traps and we are concerned about its southward progression in the coming decades.^26^ Furthermore, there is a negative synergy between air pollution and aeroallergens driving respiratory disease. This includes:

- making pollens fragile and aggravating the release of sub-pollen particles and allergenic proteins;
- the attachment of toxic particles such as heavy metals, nitrates and sulphur compounds to inhaled pollen grains which aggravate airway inflammation and weaken the mucosal barrier.^6,27,28^

PM_2.5_ also triggers oxidative stress, leading to epithelial cell damage and increased permeability, which increases allergen sensitivity and exacerbates aeroallergen-associated asthma.^25,29^

### State Of The Evidence: South African Data And Gaps

Meteorological and air-quality metrics are monitored by the South African Weather Service (SAWS) and the National Ambient Air Quality Monitoring Network (NAAQMN), respectively.^30^ They collect and analyse data through an extensive network of automatic weather stations, radars, ocean buoys, ozone spectrophotometers and air-quality monitoring stations. The National Ambient Air Quality Standards (NAAQS) define permissible levels for pollutants such as PM_10_, O_3_ and SO_2_. Real-time air-quality data, including PM_2.5_, PM_10_, CO, NO, NO_2_, SO_2_ and O_3_, are available publicly through the South African Air Quality Information System (SAAQIS).^31^ Systematic pollen and fungal-spore monitoring is coordinated by the South African Pollen Monitoring Network (SAPNET), which was established in 2019.^32^ SAPNET publishes weekly pollen counts and trends via the public platform www.pollencount.co.za. Together, these systems provide important environmental exposure data, although large swathes of communities remain unmonitored.

The impacts on health of climate-driven environmental changes are already evident. An analysis of hospital data from seven private hospitals in Cape Town (2011–2016) found that increased temperature variability was associated with a 2.5% rise in respiratory hospitalisations for males and 3% for females, with a one-day lag time.^33^ A retrospective analysis of childhood asthma admissions in 2009, 2014 and 2019 provided the first local evidence linking diurnal temperature variability to asthma exacerbations. For every 1 °C rise in day-to-night temperature difference, admissions rose by 1.4%.^34^ In a cross-sectional cohort of South African schoolchildren, household dampness and visible mould growth were associated with a 2.6-fold increase in the odds of current wheeze and a 3.4-fold increase in the odds of risk of rhinitis.^35^

Ambient air pollution remains a major concern, with local concentrations often exceeding World Health Organisation (WHO) limits by 10 to 20 times.^36^ A systematic review reported that heavy truck traffic near homes increased wheeze risk in children by 2.2 times, while proximity to mine dumps was associated with a 1.4-fold increase in wheeze and a twofold increase in chronic cough. Asthma and bronchial hyper-reactivity were linked to mean SO_2_ concentrations. Increases in PM_2.5_ and SO_2_ were significantly associated with acute respiratory symptoms and measurable declines in lung function among children, with the average five-day SO_2_ exposure linked to a reduction in peak expiratory flow rate (PEFR), and PM_2.5_ exposure associated with reduced forced expiratory volume in one second (FEV_1_) and PEFR.^37^ In contrast, a Durban study found no significant association between PM_10_ or SO_2_ levels and the prevalence of wheeze in schoolchildren.^36^ Adults are similarly affected: older adults living near a mine dump experienced a twofold increased risk of wheeze and cough. In Cape Town, an interquartile range increase of 12 µg/m^3^ in PM_10_ was associated with a 1.9% rise in respiratory admissions; and a 7.3 µg/m^3^ increase in NO_2_ correlated with a 2.3% increase in omissions.^38^ These effects peaked during warmer months, suggesting a synergistic effect of heat and pollution. Another Cape Town study confirmed this interaction where respiratory mortality rose by 2.9% per 10 µg/m^3^ increase in PM_10_ at moderate apparent temperatures and by 3.4% at high apparent temperatures. The effect of NO_2_ was even greater, with mortality increased by 8.5% at moderate temperatures and 10.9% at high temperatures.^39^

Emerging pollen data also point to important changes. SAPNET data from 2019–2021 show a shift in pollen patterns across major cities, with exotic wind-pollinated trees now dominating airborne pollen counts, whereas indigenous species have declined.^32^ This trend, driven by urban landscaping and climate change, increases the allergen loads of the urban greenspace and extends seasonal exposure. Coastal areas experience increased mould and fungal spores, reflecting South Africa’s distinct regional variability. These patterns are concerning, given the known link between aeroallergens, AR and asthma.^40^ Understanding the interplay and interaction between exposure variables is now a key area of global study, as it enables identification of cumulative and converging high-risk exposure periods, which in turn allows for the development of targeted region-specific mitigation and health strategies.

Assessing the impact of climate change on respiratory health, on both short and longer time scales, depends on consistent and sufficiently granular exposome monitoring. Unfortunately, despite growing efforts, our environmental monitoring systems are fragmented, inconsistent and uneven, with data collection concentrated in urban centres, which limits the generalisability to rural and informal settings. Geographic coverage is particularly poor for pollen and fungal spore surveillance, with only seven operational traps in place nationally, and equipment reliability across meteorological, air quality and bioaerosol platforms remaining a concern. Aeroallergen monitoring, although improving, is still insufficient to capture the full range of seasonal and regional variation in pollen and fungal exposure, with several regions of South Africa remaining unmapped.^41^ Whereas several government-supported institutions produce robust and publicly available environmental reports, the data are often not designed for academic analysis and remain underused in health-research contexts. No integrated framework links environmental exposures to respiratory health outcomes.

Granular, community-level respiratory health data are equally essential to meaningful analysis and intervention. At present, while health data collection exists across sectors, it is often limited by inconsistent data collection, multiple disconnected systems and inadequate digitisation.^42^ Practices vary widely across the country, leaving data fragmented and often inaccessible.^43^ Even digitised data are frequently not harmonised or are incorrectly coded, rendering analysis prone to error.^44^ Private-sector data, though more structured, remain largely inaccessible due to privacy restrictions and they also tend to reflect wealthier urban populations, which introduces bias and reduces the relevance across socio-economic and geographic contexts.

Research designs and access to research are additional obstacles. As a result of limited access, researchers may rely on ecological analyses using aggregated data rather than on precise individual-level assessments, reducing causal inference. Most existing South African studies are cross-sectional or short-term, often focusing on children and the elderly, with a lack of longitudinal population-level data. Research on the impact of heat and temperature variability on health, particularly in relation to asthma, is sparse. Little is known about post-flood fungal exposure or how different housing structures influence exposure risk. Overall, South Africa’s surveillance infrastructure remains fragmented and under-resourced when compared to international standards. This underscores the urgent need for coordinated context-specific research and improved systems for climate–health data integration.

### Early-Warning Systems

**Figure 2:**
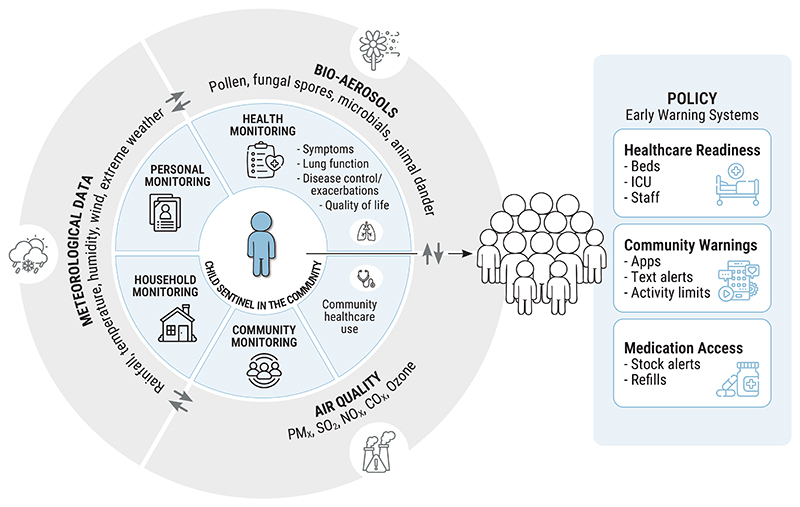
Conceptual framework of the SA-CARES sentinel surveillance system for climate-related respiratory risk

Effective early-warning systems for climate-related respiratory threats depend on robust integrated surveillance that links environmental exposures to health outcomes in real time. Existing alerts, such as those for air quality or heatwaves, are often underused and rarely linked to actionable healthcare responses. The SA-CARES project (South Africa – Climate Air-emissions and Respiratory Health Early Sensing) aims to fill this gap by implementing a sentinel surveillance system that monitors climate variables, air pollutants and bio-aerosols alongside respiratory health indicators in paediatric asthmatic sentinels, and the use of healthcare at the community level. SA-CARES represents a proactive, locally responsive model for climate – health resilience. By enabling the geographically sensitive detection of risk patterns it may support timely interventions such as clinical preparedness, community alerts and public-health messaging. If successful, it will provide a scalable framework for early response and adaptation. Figure 2 illustrates the SA-CARES conceptual framework linking environmental exposures to respiratory metrics in order to support locally tailored early-warning strategies.

### Policy And Decision-Making

Strengthening South Africa’s response to climate-related respiratory health risks requires integrated systems that link health metrics to environmental exposures. National efforts to improve data collection, digitisation, harmonisation and data-sharing, particularly in government-supported facilities, are essential to enabling accurate analysis of trends and evidence-based decision-making. Mitigation and adaptation strategies must operate at the national, provincial, community and individual levels if they are to be effective.^45^ The COVID-19 pandemic highlighted the value of accurate real-time data and the utility of cooperative data-sharing.^46^ Platforms such as the National Institute for Communicable Diseases (NICD) dashboard enabled rapid resource allocation and informed national strategy. Existing early-warning systems for air pollution, heatwaves and extreme weather would benefit from increased public use, improved visibility and integration with public-health response frameworks through implementation research and stakeholder collaboration. Community engagement is crucial to fostering empowered communities and ensuring local ownership of adaptation efforts. Translating data into action to achieve meaningful progress will require coordinated leadership, sustained investment in infrastructure and the co-creation with communities of policies that are responsive to their needs.

## Conclusion

Climate change is intensifying South Africa’s respiratory health burden through increased exposure to extreme weather, air pollution and aeroallergens. These environmental pressures interact with entrenched social and healthcare vulnerabilities, contributing to poor asthma control, elevated mortality and widening health inequities. While the health risks are increasingly being recognised, South Africa’s surveillance infrastructure remains fragmented, and consequently comprehensive long-term respiratory health data remain scarce. Improving environmental and health data systems, strengthening exposure – health linkages and resolving data silos are urgent priorities. Context-sensitive research and early-warning systems such as SA-CARES offer promising frameworks for strategic, locally relevant responses. Coordinated investment in data integration, healthcare preparedness and community engagement will be key to building adaptive capacity and advancing climate-resilient health policy in South Africa.

## References

[1] Prflss-Üstfln A, Wolf J, Corvalán CF, Bos R, Neira M (2016). Purificación. (Preventing disease through healthy environments: A global assessment of the burden of disease from environmental risks.

[2] World Meteorological Organization (2024). State of the Climate 2024: Update for COP29.

[3] Tollefson J (2025). Earth breaches 1.5 °C climate limit for the first time: what does it mean?. Nature.

[4] Department of Forestry F and the E (2024). Biennial transparency report to the United Nations Framework Convention on Climate Change under the Paris Agreement.

[5] Vicedo-Cabrera AM, Melén E, Forastiere F (2023). Climate change and respiratory health: A European Respiratory Society position statement. Eur Resp J.

[6] Dhankhar A, Darssan D, Dey S (2025). Influence of ENSO, droughts, and temperature rise on pollen and pollen seasons in Australia. Science of the Total Environment.

[7] Mafwele BJ, Lee JW (2022). Relationships between transmission of malaria in Africa and climate factors. Sci Rep.

[8] Nuttall PA (2022). Climate change impacts on ticks and tick-borne infections. Biologia.

[9] Nhamo G, Chapungu L, Mutanda GW (2025). Trends and impacts of climate-induced extreme weather events in South Africa (192032023. Environ Dev.

[10] Engelbrecht F, Wright CY, Vogel C (2022). Living with climate health risks – opportunities and challenges in southern Africa. Wits J Clin Med.

[11] Pakdehi M, Ahmadisharaf E, Azimi P (2025). Modeling the latent impacts of extreme floods on indoor mold spores in residential buildings: Application of machine learning algorithms. Environ Int.

[12] Richards G, Mcdonald M, Gray C (2023). Allergic rhinitis: Review of the diagnosis and management: South African Allergic Rhinitis Working Group. S Afr Gen Pract.

[13] Acosta-España JD, Romero-Alvarez D, Luna C, Rodriguez-Morales AJ (2024). Infectious disease outbreaks in the wake of natural flood disasters: Global patterns and local implications. Infezioni in Medicina.

[14] Oluyomi AO, Panthagani K, Sotelo J (2021). Houston hurricane Harvey health (Houston-3H) study: Assessment of allergic symptoms and stress after hurricane Harvey flooding. Environ Health.

[15] He C, Salonen H, Ling X (2014). The impact of flood and post-flood cleaning on airborne microbiological and particle contamination in residential houses. Environ Int.

[16] Makrufardi F, Manullang A, Rusmawatiningtyas D (2023). Extreme weather and asthma: A systematic review and meta-analysis. Eur Respir Rev.

[17] Zhou Y, Pan J, Xu R (2022). Asthma mortality attributable to ambient temperatures: A case-crossover study in China. Environ Res.

[18] Çelebi Sözener Z, Treffeisen ER, Özdel Öztürk B, Schneider LC (2023). Global warming and implications for epithelial barrier disruption and respiratory and dermatologic allergic diseases. J Allergy Clin Immunol.

[19] Deng L, Ma P, Wu Y (2020). High and low temperatures aggravate airway inflammation of asthma: Evidence in a mouse model. Environ Pollut.

[20] Balmes JR, Hicks A, Johnson MM, Nadeau KC (2025). The effect of wildfires on asthma and allergies. J Allergy Clin Immunol Pract.

[21] D’Amato G, Agache I, Nadeau K (2025). Wildfires and respiratory allergy. Allergy.

[22] Aguilera R, Corringham T, Gershunov A, Leibel S, Benmarhnia T (2021). Fine particles in wildfire smoke and pediatric respiratory health in California. Pediatrics.

[23] Council for Scientific and Industrial Research (CSIR) (2019). Wildfires: The impact of climate change on wildfires in South Africa.

[24] South African Environmental Observation Network (SAEON) Climatological hazards.

[25] Singh AB, Kumar P (2022). Climate change and allergic diseases: An overview. Front Allergy.

[26] Gharbi D, Berman D, Neumann F (2024). Ambrosia (ragweed) pollen – a growing aeroallergen of concern in South Africa. World Allergy Organ J.

[27] Sénéchal H, Visez N, Charpin D (2015). A review of the effects of major atmospheric pollutants on pollen grains, pollen content, and allergenicity. Scientific World Journal.

[28] Aghapour M, Ubags ND, Bruder D (2022). Role of air pollutants in airway epithelial barrier dysfunction in asthma and COPD. Eur Respir Rev.

[29] Marczynski M, Lieleg O (2021). Forgotten but not gone: Particulate matter as contaminations of mucosal systems. Biophys Rev.

[30] South African Weather Service (SAWS) Overview.

[31] Department of Forestry, Fisheries and Environment South African Air Quality Information System (SAAQIS).

[32] Esterhuizen N, Berman DM, Neumann FH (2023). The South African Pollen Monitoring Network: Insights from 2 years of national aerospora sampling (201932021). Clin Transl Allergy.

[33] Makunyane MS, Rautenbach H, Sweijd N, Botai J, Wichmann J (2023). Health risks of temperature variability on hospital admissions in Cape Town, 2011–2016. Int J Environ Res Public Health.

[34] Phakisi TK (2022). Assessing the role of temperature and air pollution in exacerbating childhood asthma in Cape Town, South Africa.

[35] Olaniyan T, Dalvie MA, Röösli M (2019). Asthma-related outcomes associated with indoor air pollutants among schoolchildren from four informal settlements in two municipalities in the Western Cape Province of South Africa. Indoor Air.

